# Analysis of public engagement with ten major global health topics on a social network profile and a newspaper website

**DOI:** 10.7189/jogh.10.010902

**Published:** 2020-06

**Authors:** Iain H Campbell, Igor Rudan

**Affiliations:** Centre for Global Health, Usher Institute for Informatics and Population Sciences, University of Edinburgh, Edinburgh, Scotland, UK

## Abstract

**Background:**

Efforts in global health and development have broad political support and substantial financial commitment from most governments. However, this support could be greater if global health issues featured more prominently in the public debate. It has proven quite difficult to make global health issues attractive for viewing and engaging with, as compared to other forms of entertainment or public debates in the media.

**Methods:**

Within the Massive Open Online Course “Survival: The Story of Global Health”, we created 10 educational videos on major global health topics. Between August 1 and September 30, 2017, we posted each episode with a brief background text on the Facebook profile of the narrator, who had an average of 450 friends and further 800 followers throughout the period of study. We studied the interaction of Facebook friends and followers with each posted video, tracing the number of their “likes”, “shares” and “comments”. Moreover, a popular Croatian online newspaper portal with about 250 000 daily viewers shared three of these stories after they were posted on Facebook and views, shares and comments were monitored. We recorded the effect on the number of YouTube views of the featured videos.

**Results:**

The 10 posts received between 65 and 274 “likes” on the Facebook profile and between 2 and 124 shares, receiving between 0 and 17 comments. The three episodes that were shared by the online newspaper portal were further shared between 164 and 2820 times, receiving between 8 and 111 comments from the general public. The effect of these two promotion channels on YouTube viewership resulted in between 107 and 9784 views of the 10 featured videos, with the number of “likes” received on YouTube ranging between 0 and 43. The video that raised the most attention and shares was the one on the history of pandemics, which also had the highest number of shares on YouTube (n=69), followed by the video on human evolution (n = 14). Topics of non-communicable diseases, ageing and dying, and the future of humanity were also popular, while the topics more specific to global health raised less interest - ie, maternal and child mortality, major infectious diseases, international organizations, inequality and equity, and the UN Millennium Development Goals.

**Conclusion:**

Our study showed that the interest in “core” global health topics was, as a rule, lower than in the topics which have a more general appeal - such as pandemic threat, human origins, ageing and dying. If we aim to increase public interest in global health topics, a feasible strategy would be to adjust the language and presentation used to be of more appeal to popular culture. Linking promotional materials to other popular topics that are dominating the public debate or capturing their interest could prove to be a successful strategy to achieve this.

Efforts in global health and development have broad political support and substantial financial commitment from most governments, but this support could be greater if global health issues featured more prominently in the public debate. However, it has proven quite difficult to make global health issues attractive for viewing and engaging with, as compared to various forms of entertainment or public debates in the media [1]. It is unclear how best to engage the public with global health topics. We recently reviewed effective approaches to public engagement and identified several success stories in conveying global health issues to wide audiences online [1,2].

The late Dr Hans Rosling was remarkably successful in visualising extremely complex and multi-dimensional data on global health, which he presented in a way that was engaging and educational for the broadest of audiences [3]. His short film “200 Countries, 200 Years, 4 Minutes - The Joy of Stats”, aired by BBC 4, has accumulated more than 9 million views on YouTube [4], while his famous TED talk “Debunking third-world myths with the best stats you've ever seen” was viewed 1.9 million times [5]. Moreover, educational videos for frontline health workers, developed by Global Health Media Project, such as the animations “The Story of Ebola” [6] and “The Story of Cholera” [7] were also viewed by millions. However, these successes are very rare outliers. There are less than 100 videos on YouTube today with a “global health” tag that have been seen by 10 000 viewers or more. At the same time, terms relevant to global health, such as “superbugs”, “antibiotic apocalypse” or “zika” can be very popular and attract millions of viewers, but they do not use scientific vocabulary and are often misleading.

To the best of our knowledge, no studies have used experimental design to determine factors that could affect the uptake of global health topics on social media. In this study, we developed a 10-episode documentary series on global health at the University of Edinburgh, which also serves as a massive open online course (MOOC). In each episode we varied the topics, presentation style and music. We then published each episode on the personal Facebook profile of the narrator (IR) and followed its further progress on social media and uptake by online news portals. It was expected that the differences between the episodes could inform strategies for promoting global health-related videos online.

## METHODS

During the first seven months of 2017 we created a documentary series on global health called “Survival: The Story of Global Health”, which also serves as a MOOC. The series contains 10 educational videos on major global health topics. Descriptive details of each episode, including a title of each episode, a brief summary of content, duration, predominant style in which the episode was made (classic documentary movie, dramatic narrative, artistic narrative or animated narrative) and the link to each episode on YouTube are available in **Table 1**.

**Table 1:** Description of the 10 episodes from the documentary series (and MOOC) entitled “Survival: The Story of Global Health”. The table provides a title of each episode, a brief summary of content, duration, predominant style in which the episode was made (classic documentary movie, dramatic narrative, artistic narrative or animated narrative) and the link to each episode on YouTube

Episode number	Title	Content summary	Duration (min:sec)	Style	YouTube link
Episode 1	How did we avoid extinction?	Survival of human species to modern times	9:10	Classic	https://www.youtube.com/watch?v=LhDoS6T6DHA&t=5s
Episode 2	Women and children first	Maternal, newborn and child health	7:57	Classic	https://www.youtube.com/watch?v=CrfxgzAi_3o&t=35s
Episode 3	Invisible friends and visible enemies	Infectious diseases, particularly malaria, tuberculosis and HIV/AIDS	15:22	Classic	https://www.youtube.com/watch?v=wo6ocbDjyeo&t=46s
Episode 4	Keeping our enemies closer	History of pandemics and new pandemic threats of the 21^st^ century	19:49	Dramatic	https://www.youtube.com/watch?v=yM-92ZYjTxQ&t=1062s
Episode 5	Our private diseases	Non-communicable diseases globally	14:47	Artistic	https://www.youtube.com/watch?v=9STZq5uAAYI&t=27s
Episode 6	Are ageing and dying inevitable?	Biological limits to human lifespan and related biotechnological advances	15:18	Artistic	https://www.youtube.com/watch?v=YAKc4N_GBvs&t=3s
Episode 7	Who rules the new world of global health?	International organizations and philanthropic foundations in global health	8:40	Classic	https://www.youtube.com/watch?v=1mCUBDrvTwM&t=19s
Episode 8	Agreeing on goals for humanity	UN's Millennium Development Goals and Sustainable Development Goals	8:50	Animated	https://www.youtube.com/watch?v=IX7PH8ofmGk&t=19s
Episode 9	Leave no-one behind	Issues related to inequality, equity and universal health coverage	9:11	Artistic	https://www.youtube.com/watch?v=u0JejcRIDA0&t=26s
Episode 10	Could we make it to the end of time?	The future of human species and the likely challenges to long-term survival	13:27	Artistic	https://www.youtube.com/watch?v=CAiq1KdgRzU&t=169s

Between August 1, 2017 and September 30, 2017 we posted each episode with a brief background text on the Facebook profile of the narrator, who had an average of 450 friends and further 800 followers throughout the period of study. We studied the interaction of Facebook friends and followers with each posted video, tracing the number of their “likes”, “shares” and “comments”. The dates of posting, the number of “likes”, “shares” and “comments” are presented in **Table 2**.

**Table 2:** The outcome of posting of each episode on personal Facebook profile, which took place between 1 August and 5 October 2017

	Episode 1	Episode 2	Episode 3	Episode 4	Episode 5	Episode 6	Episode 7	Episode 8	Episode 9	Episode 10
Date posted on FB	26 Aug 17	29 Aug 17	01 Aug 17	05 Aug 17	01 Sep 17	19 Sep 17	14 Sep 17	18 Sep 17	21 Sep 17	22 Sep 17
Likes on FB	274	171	65	226	246	143	112	95	123	89
Shares from FB	57	16	2	124	34	11	11	11	18	2
Comments on FB	14	10	0	12	17	9	4	0	11	4
Picked by ONP	Yes (1)	No	No	Yes (3)	Yes (1)	No	No	No	No	No
Shared from ONP	164	0	0	2820	257	0	0	0	0	0
Comments on ONP	8	0	0	111	29	0	0	0	0	0
Views on YT (5 d)	1263	108	123	9784	270	201	166	135	171	107
Likes-dislikes on YT	21-0	0-0	3-0	43-2	4-0	12-0	0-0	2-0	3-0	3-0
Shares from YT	14	0	1	69	2	1	0	1	4	3
Subscribers on YT	5	0	2	20	0	0	0	1	0	0
Average view on YT	2:58	2:54	3:54	4:44	2:38	4:48	2:41	2:47	3:12	4:02
Viewed 1 min on YT	48%	42%	42%	51%	38%	48%	43%	53%	47%	36%
Peaks in view on YT	1	1	2	0	2	1	0	2	1	3
Content related to each peak in view on YT	Narrator appears	Script begins	Malaria cycle; TB treatment	None	Food and BMI; Franken-stein pub	CRISPR	None	Global agenda; Poverty reduction	Inequality agenda	Narrator appears

FB – Facebook, ONP – Online News Portal, YT – You Tube, TB – tuberculosis, BMI – body mass index, CRISPR – gene-editing tool in molecular biology

Moreover, several popular Croatian online news portals (Telegram.hr, Index.hr and Liberal.hr) with between 100 000 and 500 000 daily viewers chose to share some of these stories from those public Facebook posts. Their websites tracked the number of views, shares and comments, which we monitored and recorded (**Table 2**). The resulting effects of those interactions on YouTube views of the featured videos were recorded and analysed using YouTube Analytics (**Table 2**). We recorded the number of YouTube views five days after the initial Facebook post. We also documented the number of “likes” and “dislikes” received for each YouTube video, and the number of subscribers to the YouTube channel “Survival: The Story of Global Health” that each of the 10 videos attracted. We also recorded the average viewing duration for each video, retention of viewers after 1 minute of each episode, number of shares on YouTube, number of peaks in the viewership over the duration of each episode, and the content that most likely caused the peak (**Table 2**).

## RESULTS

**Table 1** provides information on the 10 episodes related to global health topics that we developed. The 10 themes covered by these episodes were: (i) Survival of human population to modern times; (ii) Maternal, newborn and child health; (iii) Infectious diseases, particularly malaria, tuberculosis and HIV/AIDS; (iv) History of pandemics, epidemics and new pandemic threats of the 21^st^ century; (v) Non-communicable diseases; (vi) Ageing and dying; (vii) International organizations and philanthropic foundations in global health; (viii) UN's Millennium Development Goals and Sustainable Development Goals; (ix) Issues related to inequality, equity and universal health coverage; and (x) The future of global health and the likely challenges to long-term survival. The duration of these episodes ranged from 7 minutes 57 seconds (Episode 2) to 19minutes 49 seconds (Episode 4).

To gather as much information as possible from this experiment, we also varied the styles in which different episodes were produced: four episodes (numbers 1, 2, 3 and 7) were produced in the style of a classical documentary movie; four episodes (numbers 5, 6, 9 and 10) in the more artistic style; one episode (number 4) was made to appear rather dramatic, while one episode (number 8) was mostly animated. This gave us a range of combinations of content, episode duration and production styles that we could analyse for their success in being shared online.

**Table 2** shows that we posted the episode on a personal Facebook profile with an average of 450 friends and 800 followers during the period of study, ie, between 1 August and 5 October 2017. The 10 Facebook posts were liked between 67 and 258 times on the Facebook profile, and shared between 3 and 123 times, receiving between 0 and 27 comments. Nearly all of the comments were very supportive and complimentary, but there was a clear difference between uptake rates for different episodes.

Three episodes clearly stood out in terms of popularity on Facebook: Episode 1 on the survival of the human species (274 likes and 57 shares), Episode 4 on historic and modern pandemics (226 likes and 124 shares) and Episode 5 on non-communicable diseases (246 likes and 34 shares). Those levels of interest trigged “detection of viral content” tools at the three online news portals in Croatia: Telegram.hr (around 250 000 visits per day), Index.hr (more than 750 000 visits per day) and Liberal.hr (around 100 000 visits per day). The first one, Telegram.hr, engaged with all three posts and turned them into a story on their news portal. Index.hr and Liberal.hr only engaged with Episode 4, which clearly stood out in terms of interest from mainstream online media.

Once the three episodes were featured by online media portals, the number of their views and shares increased quite dramatically in comparison to Facebook-based promotion alone. From the position where only 1250 friends and followers could theoretically engage with the content, exposure of three episodes in the online news portals increased the number of potential viewers to 250 000 for Episodes 1 and 5, and to more than 1 million for Episode 4. Tracking of interaction with the story showed that Episode 1 was shared on Facebook 164 times (in addition to 57 original shares) and raised 8 further comments; Episode 5 was shared 257 times (in addition to original 34) and raised 29 further comments; while Episode 5 was shared 2820 times (in addition to original 124) and raised 111 further comments (**Table 2**).

All of these developments led to viewing of the 10 videos on YouTube, which we also recorded and analysed. As expected, the seven episodes that weren't further shared by the online news portals and which had Facebook as their only means of dissemination (ie, episodes 2-3 and 6-10) gained between 107 and 201 YouTube views. This number is typically about 30%-50% larger than the number of likes that each Facebook post received, which means that the number of actual viewers was larger than the number of those who liked the Facebook post. This observation created a consistent base for expected interaction from a Facebook post. However, the impact of the remaining three episodes, which were further shared by online news portals, were different: Episode 5 received 270 YouTube views, Episode 1 received 1263 views, and Episode 4 received as many as 9784 views in 5 days (**Table 2**).

The most viewed episode (Episode 4, on pandemics) also attracted most “likes” on YouTube (n = 43) and most “shares” (n = 69), but also 2 “dislikes”, which were the only such cases. No other episodes received a dislike on YouTube. The number of “likes” was also quite high for Episode 1 on human evolution (n = 21), which also had 14 “shares” from YouTube, while the Episode 6 on ageing and dying received 12 “likes” and only 1 “share”. Other episodes received between 0 and 4 “likes” and 0 to 4 “shares”. In terms of attracting new subscribers to the YouTube channel “Survival: The Story of Global Health”, Episode 4 attracted 20 subscribers, Episode 1 attracted 5 subscribers, while all other episodes combined have only managed to attract 3 subscribers (**Table 2**).

The average viewing time on YouTube ranged from 2:41 minutes (for Episode 7 on international organisations) to 4:48 minutes (for Episode 6 on ageing and dying). Episode 4 on pandemics also did better than most other episodes, with an average viewing time of 4:44 minutes, thus very nearly topping the list. However, a clear message from this analysis is that the average viewing time was much shorter than the total duration of each episode and that the drop-out rate during the course of the video might be quite high. This finding prompted further analyses to explore the patterns of viewers' dropping out. It was shown that between 36% and 53% of viewers are still present at the end of the first minute of each video, meaning that 47%-64% of all viewers would drop out within the first minute (**Table 2**). We also identified peaks in viewership at specific times in an episode. The number of peaks ranged from 0 to 3 per episode. They were related to narrator appearing, script starting, malaria parasite cycle, treatment for tuberculosis, CRISPR technology, link between food and BMI, agenda on poverty reduction and inequality.

Overall, the 4^th^ episode clearly raised the most attention, views and shares. It was the episode on the history of pandemics, which was the only episode filmed in a dramatic style, and also the longest episode. The topics that did not raise much interest were those more specific to global health – maternal and child mortality, major infectious diseases, international organizations, equity and Millennium Development Goals.

## DISCUSSION

Our study provided some early insights into determinants of public interest in global health topics on the internet. The diversity of topics addressed in 10 videos was sufficient to observe true differences in attention that were generated by the shared videos. Three of the videos managed to raise sufficient attention to be shared further by a newspaper online portal, while one of them was shared by three newspaper online portals. The other seven videos generated much less interest: they only managed to receive around 60 to 170 “likes” on Facebook and around 100 to 200 views of the video on YouTube. However, the “viral” video on pandemics and epidemics reached nearly 10 000 views, which is 50-100 times more interest than the level expected for most videos.

This experiment showed that the interest in “core” global health topics was, as a rule, lower than in the topics which have a more general appeal to the public, such as pandemic threat, human origins, or non-communicable diseases. If we aim to increase interest in and attractiveness of global health topics to the general public, a feasible strategy would be to adjust the language that is used to be of more appeal to popular culture. Linking promotional materials to other popular topics that are dominating the public debate or capturing their interest could prove to be a successful strategy to achieve this.

To contextualise this study and understand its generalizability, we should first stress that it was conducted among the 1200 Croatian followers of the narrator of the videos (IR). At the time, the narrator was already a well-known researcher and a leading public intellectual in Croatia, so it was expected that his sharing of the videos will receive some attention. Moreover, his followers were mostly highly educated persons, ie, an audience more receptive to documentary content than the general population. It is reasonable to assume that these two factors assisted the initial uptake and spread of the videos in the online space. All 10 videos were shared at the same time of the day, accompanied by a brief text describing their content, to ensure that those two factors do not affect the uptake and initial interest.

Taking into account these baseline considerations, it is clear that the differences in interest in 10 videos among the audience were significant. Three videos stood out and attracted further attention from one online newspaper portal (Telegram.hr), while one of them attracted attention from three online newspaper portals (Telegram.hr, Index.hr and Liberal.hr) and went “viral”, receiving nearly 10 000 views in the two following days. Unlike all other videos, the style of this particular video was described as “dramatic”. Also, the video was well aligned with an ongoing public debate in Croatia about the need for vaccination of children.

Several general lessons can be drawn from this experiment. First, it is incredibly difficult to make many “core” topics in global health - such as maternal and child health, infectious diseases or equity – “attractive” and interesting to viewers, even when they are presented by well-known public intellectuals to a receptive audience. Second, to achieve some uptake, viewers need to find the content particularly interesting (as in the case of Episode 1 on human population survival) or useful to them personally (as in the case of Episode 5 on non-communicable diseases).

Only in exceptionally rare cases will a combination of a well-known presenter and receptive audience lead to a “viral” spread of global health-related content online. In the case of Episode 4, we explain this additional interest through a combination of a “doomsday” topic (pandemics) and the presence of a controversy and an ongoing public debate (antivaxxers, ie, groups of people who question the value of vaccines). Only through a combination of all those enhancing factors did this video dominate the online news space in Croatia for 48 hours. Another factor that could have contributed to interest of online newspaper portals to as many as three of these videos was the timing of release during the two months of summer break, when news on politics or sport events do not dominate media space as they do throughout the rest of the year. These different factors all combined to generate large public interest in a global health topic in Croatia. On the other hand, the videos on topics more specific to global health simply weren't able to attract as much interest or attention of general public, which is an important lesson in itself.

How can these findings be used to increase public interest in global health topics through online campaigns? It is conceivable that they point to a strategy of linking global health messages to popular debates or controversies in the media which are drawing large audiences. This would clearly require a lot of creativity and perhaps insights from commercial marketing strategy will prove useful in designing such campaigns.
